# Increased intracellular H_2_S levels enhance iron uptake in *Escherichia coli*

**DOI:** 10.1128/mbio.01991-24

**Published:** 2024-09-26

**Authors:** Shouta Nonoyama, Shintaro Maeno, Yasuhiro Gotoh, Ryota Sugimoto, Kan Tanaka, Tetsuya Hayashi, Shinji Masuda

**Affiliations:** 1 Department of Life Science and Technology, Tokyo Institute of Technology, Yokohama, Japan; 2 Department of Biological Chemistry, College of Agriculture, Yamaguchi University, Yamaguchi, Japan; 3 Department of Bacteriology, Faculty of Medical Sciences, Kyushu University, Fukuoka, Japan; 4 Laboratory for Chemistry and Life Science, Institute of Innovative Research, Tokyo Institute of Technology, Yokohama, Japan; University of California, Irvine, Irvine, California, USA; New York University School of Medicine, New York, New York, USA

**Keywords:** hydrogen sulfide, persulfide, reactive sulfur species, supersulfide, YgaV, *Escherichia coli*

## Abstract

We investigated the impact of intracellular hydrogen sulfide (H_2_S) hyperaccumulation on the transcriptome of *Escherichia coli*. The wild-type (WT) strain overexpressing *mstA*, encoding 3-mercaptopyruvate sulfur transferase, produced significantly higher H_2_S levels than the control WT strain. The *mstA*-overexpressing strain exhibited increased resistance to antibiotics, supporting the prior hypothesis that intracellular H_2_S contributes to oxidative stress responses and antibiotic resistance. RNA-seq analysis revealed that over 1,000 genes were significantly upregulated or downregulated upon *mstA* overexpression. The upregulated genes encompassed those associated with iron uptake, including siderophore synthesis and iron import transporters. The *mstA*-overexpressing strain showed increased levels of intracellular iron content, indicating that H_2_S hyperaccumulation affects iron availability within cells. We found that the H_2_S-/supersulfide-responsive transcription factor YgaV is required for the upregulated expression of iron uptake genes in the *mstA*-overexpression conditions. These findings indicate that the expression of iron uptake genes is regulated by intracellular H_2_S, which is crucial for oxidative stress responses and antibiotic resistance in *E. coli*.

**IMPORTANCE:**

H_2_S is recognized as a second messenger in bacteria, playing a vital role in diverse intracellular and extracellular activities, including oxidative stress responses and antibiotic resistance. Both H_2_S and iron serve as essential signaling molecules for gut bacteria. However, the intricate intracellular coordination between them, governing bacterial physiology, remains poorly understood. This study unveils a close relationship between intracellular H_2_S accumulation and iron uptake activity, a relationship critical for antibiotic resistance. We present additional evidence expanding the role of intracellular H_2_S synthesis in bacterial physiology.

## INTRODUCTION

Hydrogen sulfide (H_2_S) is a toxic gas for cells but is also known to function as a second messenger in mammals and bacteria, participating in a wide variety of intracellular and extracellular activities, such as oxidative stress responses (1, 2). In *Escherichia coli*, 3-mercaptopyruvate sulfur transferase (MstA) is responsible for bulk of endogenous H_2_S synthesis from L-cysteine (3). Intracellular H_2_S reacts with free iron (Fe^2+^), preventing the Fenton reaction where Fe^2+^ reacts with H_2_O_2_ to produce hydroxy radicals (4). Since hydroxy radicals can damage cellular components such as proteins, lipids, and nucleic acids, MstA-dependent H_2_S production contributes to the oxidative stress response and antibiotic resistance. In *Vibrio cholerae*, intracellular H_2_S activates iron uptake and the sequestration of free ferric iron by iron storage proteins, thereby stimulating catalase activity for oxidative stress resistance (5). However, the mechanism by which intracellular H_2_S activates iron uptake remains unknown.

H_2_S is also involved in metabolism of more oxidized sulfane sulfur species known as reactive sulfur species or supersulfides (6). Supersulfides such as glutathione persulfide (GSSH) and cysteine persulfide (CysSSH) also contribute to antibiotic resistance and oxidative stress response in bacteria (7–11). Specifically, supersulfides modify specific cysteine residues of the MarR-type transcription factor to derepress the expression of its target genes, including *marA*, although the transcription factor activity is influenced by a wide variety of redox stressors. Expressed MarA upregulates the transcription of antibiotic resistance genes, such as those encoding multi-drug efflux transporters (8, 12). In the α-proteobacterium *Hyphomicrobium denitrificans*, the redox-responsive transcription factor SoxR can sense supersulfides and controls the expression of genes involved in sulfur oxidation, such as the *sox* genes, through polysulfide-dependent cysteine modification (13). Supersulfides also directly inactivate β-lactam antibiotics, thereby enhancing antibiotic resistance (14). However, intracellular H_2_S-dependent signaling network for oxidative stress response, redox balance, and antibiotic resistance is not fully understood.

The identified bacterial transcriptional regulators that specifically respond to H_2_S and/or supersulfide are classified into three categories based on structural differences: the arsenic repressor (ArsR), copper-sensitive operon repressor, and Fis superfamilies (15–23). We previously identified and characterized the ArsR-type H_2_S-/supersulfide-responsive transcription factor SqrR from the purple photosynthetic bacterium *Rhodobacter capsulatus*, which can grow photoautotrophically with H_2_S used as an electron donor (19). SqrR controls gene expression in response to H_2_S availability, crucial for photoautotrophic growth and sulfur metabolisms (24). Specifically, two conserved cysteine residues form an intramolecular tetrasulfide bond by reacting with supersulfides when extracellular H_2_S is available, leading to derepression of photosynthetic gene expression (19, 25). Interestingly, homologs of SqrR are widely conserved in non-sulfur bacteria, including *V. cholerae* and *E. coli*. The SqrR homolog of *V. cholerae*, HlyU, attenuates hemolysin transcriptional activation in response to supersulfides (26). The SqrR homolog of *E. coli*, YgaV, has also been characterized, demonstrating that YgaV represses transcription of anaerobic respiratory genes in the absence of extracellular sulfide. When sulfide concentration increases, YgaV forms an intramolecular tetrasulfide bond through supersulfides to control target gene expression (11). Both SqrR and YgaV also bind heme, potentially influencing their DNA-binding affinities, although the affinity is relatively weak (11, 27). YgaV-dependent transcriptional control prevents reactive oxygen species production, contributing to oxidative stress response and antibiotic resistance (11). Although the involvement of H_2_S, supersulfides, Fe^2+^, and some transcription factors for antibiotic resistance in *E. coli* is beginning to emerge, exact relationships between them remain elusive.

In this study, we investigated the effects of intracellular H_2_S hyperaccumulation on *E. coli* physiology, particularly antibiotic resistance. Specifically, we overexpressed *mstA* to enhance H*_2_*S production in both the *E. coli* wild type (WT) and the *ygaV* mutant (*ΔygaV*). RNA-seq analysis revealed upregulation of genes associated with drug efflux pumps in the *mstA*-overexpressing WT strain, suggesting a potential mechanism by which intracellular H_2_S contributes to antibiotic resistance. Notably, genes involved in iron uptake, including siderophore synthesis and iron import transporters, exhibited heightened expression in the *mstA*-overexpressing strain. Furthermore, our findings demonstrated that YgaV is essential for the upregulated expression of iron uptake genes in response to intracellular H_2_S accumulation. These results indicate that YgaV modulates iron uptake activity at the transcriptional level in response to intracellular H_2_S levels, a process necessary for antibiotic resistance in *E. coli*.

## RESULTS

### Overexpression of *mstA* results in hyperproduction of H_2_S

To investigate the role of intracellular H_2_S as a signaling molecule, we constructed WT *E. coli* carrying the *mstA*-overexpression plasmid pMstA. The *mstA* gene was cloned into the high-copy plasmid pUC18, which contains the ampicillin resistance marker gene, and was expressed under the control of the *lac* promotor. Even in the absence of isopropyl β-D-thiogalactopyranoside, the relative *mstA* mRNA level in the WT strain harboring pMstA, compared with those in the WT strain carrying the empty vector (pUC18), was 749 ± 24 (*n* = 3) as determined by quantitative real-time PCR (qRT-PCR), confirming successful *mstA* overexpression.

**Fig 1:**
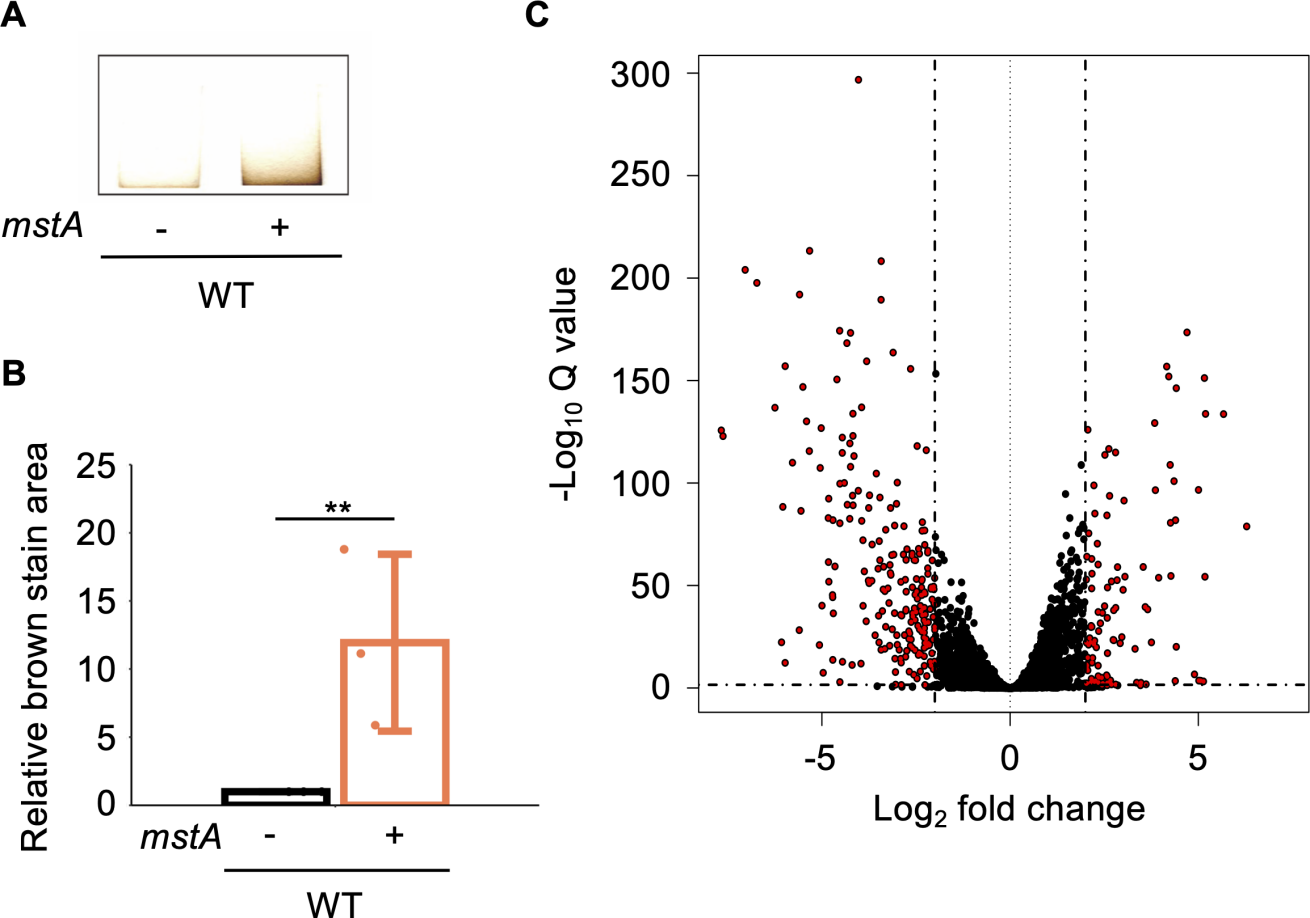
Phenotype of the *mstA-*overexpressing strain. (**A**) Brown staining on Pb(Ac)_2_ soaked papers reflecting H_2_S synthesis in the WT strain harboring an empty vector (−) and/or the *mstA*-overexpression plasmid pMstA (+). (**B**) Quantification of brown-stained areas on Pb(Ac)_2_-soaked papers shown in panel **A**. The values are means ± SDs of three biological replicates. **P < 0.05, Welch’s *t*-test. (**C**) Volcano plot for upregulated and downregulated transcripts in the *mstA*-overexpression strain compared with those in WT harboring pUC18. Red dots represent transcripts within the significance threshold (*P* < 0.05, *n* = 3).

The H_2_S production of the WT strain carrying pMstA was assessed on Pb(Ac)_2_-soaked paper, where H_2_S levels were quantified by measuring the brown-stained area (Fig. 1A). The results indicated that the WT harboring pMstA exhibited higher H_2_S production compared with the control WT strain carrying pUC18 (Fig. 1B). This finding aligns with a previous study demonstrating that overexpression of *mstA* leads to hyperproduction of H_2_S in *E. coli* (4). We utilized this strain for subsequent experiments to examine the effects of H_2_S overproduction on *E. coli* physiology.

Since H_2_S accumulation increases resistance to antibiotics (3), the presence of antibiotics (ampicillin) in the medium may affect H_2_S metabolism. When H_2_S production assays were performed without ampicillin, the WT strain harboring pMstA still produced higher amounts of H_2_S than the control strain (Fig. S1). Although the effect of antibiotics on H_2_S metabolism may still persist to some extent, these results promoted us to add ampicillin to the medium in further experiments described below.

### Impact of the H_2_S overproduction on antibiotic resistance

**Fig 2:**
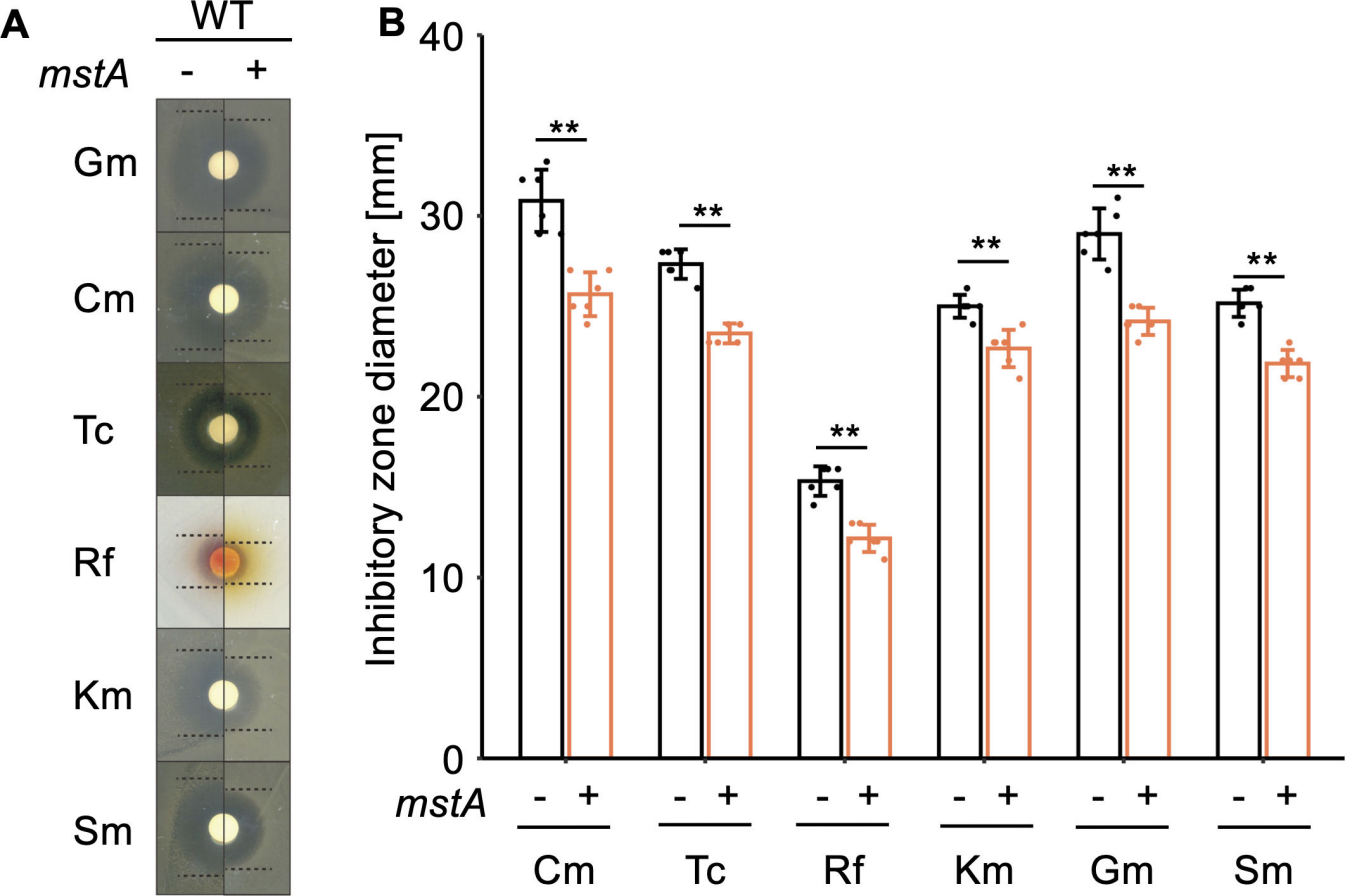
Antibiotic resistance of the *mstA-*overexpressing strain. (**A**) Photographs depicting the growth inhibitory circles around the antibiotic-soaked paper of the WT strain harboring an empty vector (−) and/or the *mstA*-overexpression plasmid pMstA (+). Dotted lines indicate the boundary of growth inhibitory circles. (**B**) Diameters of inhibitory circles shown in panel **A**, reflecting the antibiotic resistance of each strain. The values are means ± SDs of three biological replicates. **P < 0.05, Welch’s *t*-test.

We first examined the effects of H_2_S overproduction on antibiotic resistance using a disk assay, where filter paper saturated with various antibiotics was placed on *E. coli* grown plates. Resistance to each antibiotic was assessed by measuring the size of the growth-inhibited zone. As shown in Fig. 2, the H_2_S-overproducing strain (WT harboring pMstA) exhibited significantly higher resistance to all antibiotics compared with the control strain. This observation strongly suggests that intracellular H_2_S production contributes to increased antibiotic resistance. Importantly, this finding aligns with a previous report demonstrating that knockout of *mstA* results in lower H_2_S production and reduced resistance to antibiotics (3).

### Impact of the H_2_S overproduction on gene expression

Next, we conducted RNA-seq analysis to identify genes whose transcription was influenced by H_2_S overproduction. WTs carrying pMstA or the empty vector (pUC18) were cultured in Luria-Bertani (LB) medium until reaching the mid-log phase, and RNAs extracted from these cultures were subjected to RNA-seq (for details, see Materials and Methods). The obtained results showed that in the WT harboring pMstA, the transcript levels of 550 genes were significantly increased by more than 2.0-fold, while the transcript levels of 645 genes were significantly decreased by less than 0.5-fold (Fig. 1C; Data Set S1). These results indicate that intracellular H_2_S has a substantial impact on transcription of various genes in *E. coli*.

The transcript levels of most genes in cysteine metabolism, such as *metB* and *metC*, encoding cystathionine γ-lyase and cystathionine β-lyase, respectively, were not altered in the *mstA*-overexpressing strain compared to those in the WT harboring the empty vector (Data Set S1). This suggests that overexpression of *mstA* did not significantly affect the transcriptional regulation of cysteine metabolism. On the other hand, the transcript levels of *tcyP*, encoding the L-cystine transporter, increased by approximately 10-fold due to *mstA* overexpression (Data Set S1), suggesting that H_2_S overproduction by *mstA* overexpression causes cysteine depletion, which was compensated by uptake from the extracellular environment.

Of particular note, the cysteinyl-tRNA synthase (*CARS*) gene was significantly upregulated in the H_2_S-overproducing strain (Table S1). Previous studies have shown that CARS catalyzes the synthesis of various supersulfides, such as CysSSH, both *in vivo* and *in vitro* (28), suggesting a potential impact of MstA-dependent H_2_S production on CARS-dependent supersulfide synthesis. Given that supersulfides directly inactivate β-lactam antibiotics (14), the increased expression of CARS may contribute to the enhanced antibiotic resistance of the strain (Fig. 2). Importantly, as other amino-acyl tRNA synthases were also upregulated (Table S1), it suggests that the increased expression of *CARS* is not specific among amino-acyl tRNA synthase genes.

Transcript levels of several antibiotic efflux pumps, such AcrAB (29), MdtABC (30), and EmrAB (31), were also increased in the *mstA*-overexpression strain, although the differences in transcript levels between the strains ranged from two- to threefold (Table S2). The increased expression of these efflux pumps may contribute to the increased antibiotic resistance observed in response to H_2_S overproduction.

Genes involved in TonB-dependent iron uptake were also upregulated in the *mstA*-overexpression strain (Table S3). These genes are involved in the recognition and transportation of catecholate siderophores (*fepABCDG*, *fiu*, and *cirA*), hydroxamate siderophores (*fhuABCDEF*), and diferric citrate (*fecABCDE*) (32, 33). Previous studies demonstrated that intracellular H_2_S reacts with free iron to produce FeS (4) and/or upregulates sequestration of free iron by iron storage proteins (5), suggesting that the higher production of H_2_S in the strain results in free iron deficiency within cells at least transiently. As a result, the strain might be compelled to activate its iron uptake system.

Genes downregulated in the *mstA*-overexpression strain include those involved in some central metabolic pathways such as tricarboxylic acid cycle and β-oxidation (Data Set S1), suggesting that hyperaccumulation of H_2_S affects energy conversion activity in cells. In particular, several transporter genes, including a dipeptide/heme transporter (*dppA*, *dppB*, *dppC*, *dppD*, and *dppF*) and a heme transporter across the cytoplasmic membrane (c*cmA*, *ccmB*, *ccmC*, and *ccmD*) were downregulated in the *mstA*-overexpression strain (Data Set S1). DppA has been shown to transport not only dipeptide but also heme across the inner membrane (34), suggesting that hyperaccumulation of H_2_S affects heme homeostasis.

### Intracellular H_2_S activates iron uptake through YgaV-dependent transcription control

The observation that the *mstA-*overexpressing WT strain exhibited increased expression of genes involved in iron uptake suggested that excess H_2_S synthesis leads to free-iron deficiency as mentioned above. Iron deficiency is known to be sensed by the transcriptional regulator ferric uptake regulator (Fur), which has been extensively studied for more than 40 years (35). These studies have shown that under iron-rich conditions, Fur binds iron and represses the transcription of iron uptake genes. However, upon iron deficiency, iron was released from Fur, and apo-Fur derepresses its target gene expression with activating a few genes (35). Based on this assumption, we anticipated that the Fur regulon could be upregulated in the *mstA*-overexpression strain. To confirm this hypothesis, we compared genes affected by *mstA* overexpression with those regulated by Fur. A previous study identified 81 genes directly regulated by apo- and holo-Fur (35). However, two of the genes (*efeU_2* and *ryhB*) are not annotated in the WT strain (BW25113) used in this study. Therefore, we compared the remaining 79 Fur-regulated genes with our RNA-seq data.

**Fig 3:**
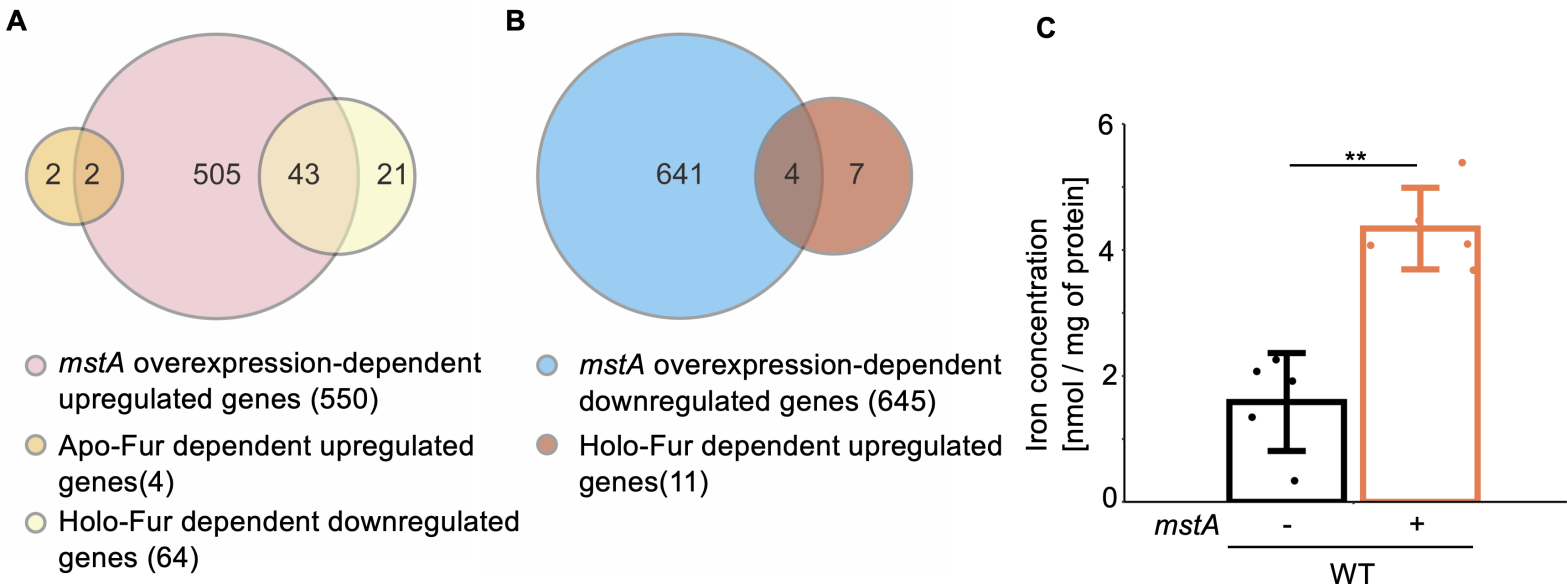
Relationship between H_2_S overaccumulation-dependent and Fur-dependent transcript changes. Venn diagrams depict the overlap between genes upregulated (**A**) and downregulated (**B**) by *mstA* overexpression, and those regulated by apo- and holo-Fur. Genes directly regulated by apo- and holo-Fur, as reported previously (35), were used for the comparison, except for *efeU_2* and *ryhB*, for which information was not found in the reference genome of the WT strain (BW25113) used in this study. (**C**) Intracellular iron content in WT harboring an empty vector (−) and/or the *mstA*-overexpression plasmid pMstA (+). The values are means ± SDs of five biological replicates. **P < 0.05, Welch’s *t*-test.

Venn diagrams revealed that the genes upregulated in the *mstA-*overexpression strain did not significantly overlap with those of the Fur regulon (Fig. 3). For instance, Fur-regulated genes like *suf*-operon and *feo*-operon genes were not upregulated in the *mstA-*overexpression strain, although genes related to iron uptake, such as *cirA* and *fes*, were upregulated in the strain. Specifically, only 45 genes out of the Fur-regulated 68 genes were upregulated (Fig. 3A), and four genes out of the remaining 11 genes were downregulated in the *mstA*-overexpressing strain (Fig. 3B). These results strongly suggest that a transcription factor other than Fur activates iron uptake genes in response to H_2_S. We performed a colorimetric ferrozine-based assay (36) with the soluble fraction of the cell-free extract of the *mstA*-overexpressing strain. The results showed that concentration of the intracellular iron including iron-containing proteins and small molecules in the *mstA*-overexpression strain was significantly higher than those in the control strain (Fig. 3C), supporting the hypothesis that the contribution of Fur to the H_2_S-dependent transcriptional upregulation, if any, was small.

**Fig 4:**
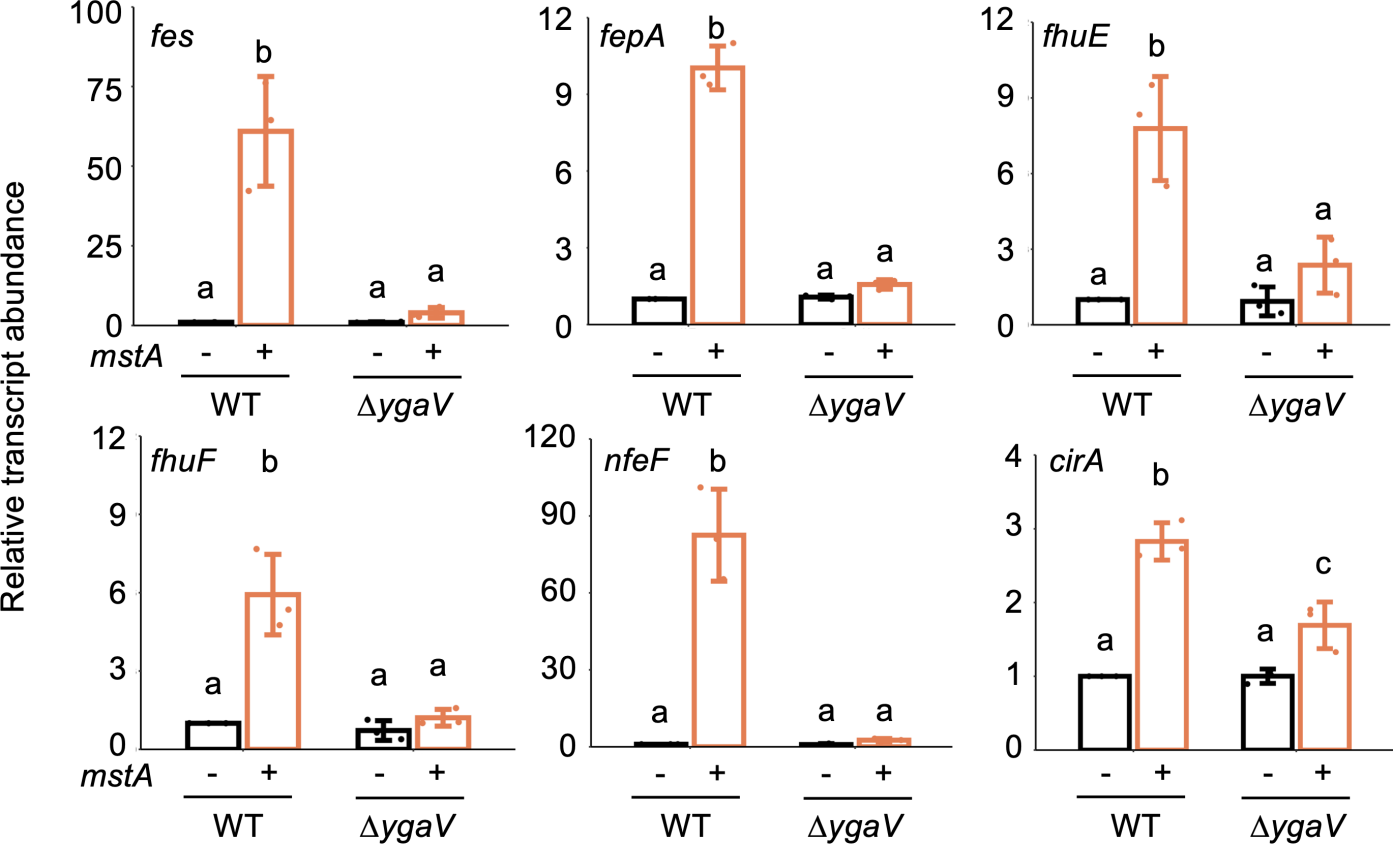
YgaV-dependent regulation of iron-uptake genes. Transcript levels of each gene were quantified by qRT-PCR. Total RNAs were extracted from the WT and/or Δ*ygaV* mutant harboring an empty vector (−) and the *mstA*-overexpression plasmid pMstA (+). The values are means ± SDs of three biological replicates. Different letters (a, b, and c) indicate a significant difference (*P* < 0.05, Tukey’s test).

We assumed that the H_2_S-/supersulfide-responsive transcription factor YgaV might be the one activating iron uptake genes in the *mstA-*overexpression strain, given that YgaV is involved in oxidative stress responses and antibiotic resistance (11). To investigate how YgaV controls the iron uptake genes upregulated in the *mstA-*overexpression WT, we quantified transcript levels of iron uptake genes in the Δ*ygaV* mutant harboring the *mstA-*overexpression plasmid (pMstA) or the empty vector (pUC18). We analyzed the transcript levels of *fes*, *fepA*, *fhuE*, *fhuF*, *nfeF*, and *cirA* in both WT and Δ*ygaV* strains in the absence and presence of pMstA. Fes and FepA are involved in siderophore enterobactin synthesis and its receptor, respectively. FhuE and FhuF are part of the siderophore reduction system and siderophore-iron reductase. NfeF is a Ni-responsive Fe-uptake flavoprotein, while CirA serves as a siderophore-binding colicin receptor. Transcript levels of all tested genes did not increase in the Δ*ygaV* harboring pMstA (Fig. 4). Additionally, the transcript levels of these genes in Δ*ygaV* strains carrying pMstA or pUC18 were comparable to those in WT carrying pUC18 (Fig. 4). These results indicate that YgaV activates transcription of iron uptake genes in response to intracellular H_2_S concentration.

### YgaV binds to the promoters of iron uptake genes

Next, we conducted modified chromatin-immunoprecipitation quantitative-PCR (ChIP-qPCR) analysis to directly assess the binding of YgaV to the promoters of iron uptake genes. We inserted a His-tag sequence at the 5′ end of the endogenous *ygaV* gene to ensure that the copy number of His-tagged YgaV in the strain remained consistent with that of the WT (for details, see Materials and Methods). Following protein–DNA crosslinking, His-tagged YgaV was isolated and purified using Ni-charged resin and subsequently subjected to qPCR analysis to measure the levels of promoter DNA for iron uptake genes. Additionally, we quantified *rrnB* DNA levels as a negative control. Furthermore, purification of His-tagged YgaV was carried out using Ni-uncharged resin for a negative control experiment.

**Fig 5:**
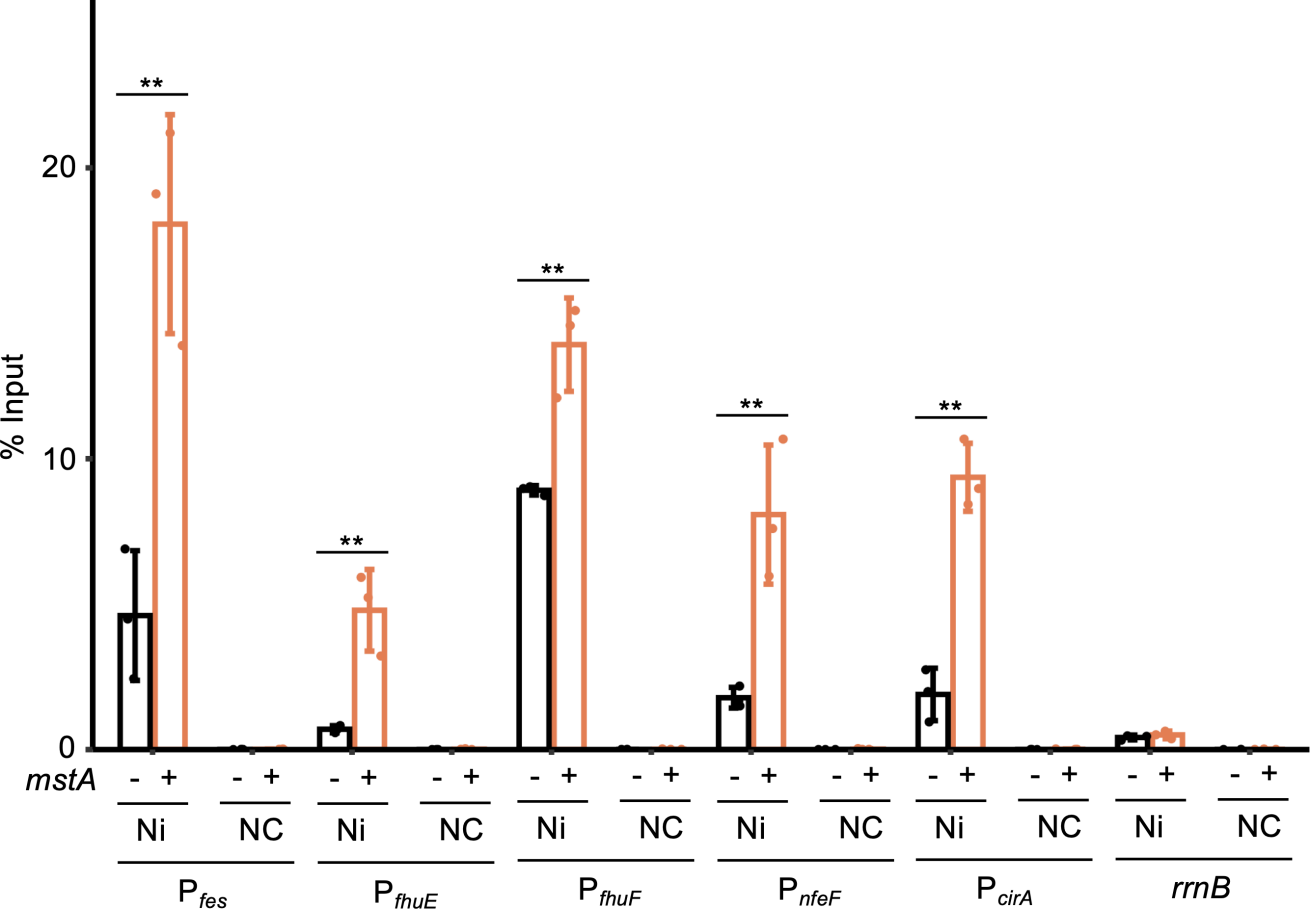
H_2_S-dependent YgaV binding to the target promoters as examined by ChIP-qPCR analysis. Binding of the His-tagged YgaV to the promoters of iron uptake genes (*fes*, *fhuE*, *fhuF*, and *nfeF*) in WT harboring an empty vector (−) and/or the *mstA*-overexpression plasmid pMstA (+) was examined as elaborated in Materials and Methods, with *rrnB* serving as a negative control gene. Pull-down of His-tagged YgaV for ChIP-qPCR was performed using a Ni-charged resin (Ni), as well as Ni-uncharged resin for a negative control (NC). The values are means ± SDs of three biological replicates. **P < 0.05, Welch’s *t*-test.

As shown in Fig. 5, DNA levels of all tested promoters, such as those of *fes*, *fhuE*, *fhuF*, *nfeF*, and *cirA*, were quantifiable when His-tagged YgaV was pulled down with Ni-charged resin (Ni), but not with Ni-uncharged resin (negative control), suggesting that YgaV binds to these promoters. More notably, the DNA levels of all iron uptake genes’ promoters examined, but not *rrnB*, were significantly higher in the strain carrying the *mstA*-overexpression plasmid pMstA (+) compared with the strain harboring the empty vector (−). These results suggest that the binding of YgaV to the promoters is augmented when intracellular H_2_S levels are increased.

### Impact of H_2_S overproduction of iron uptake

**Fig 6:**
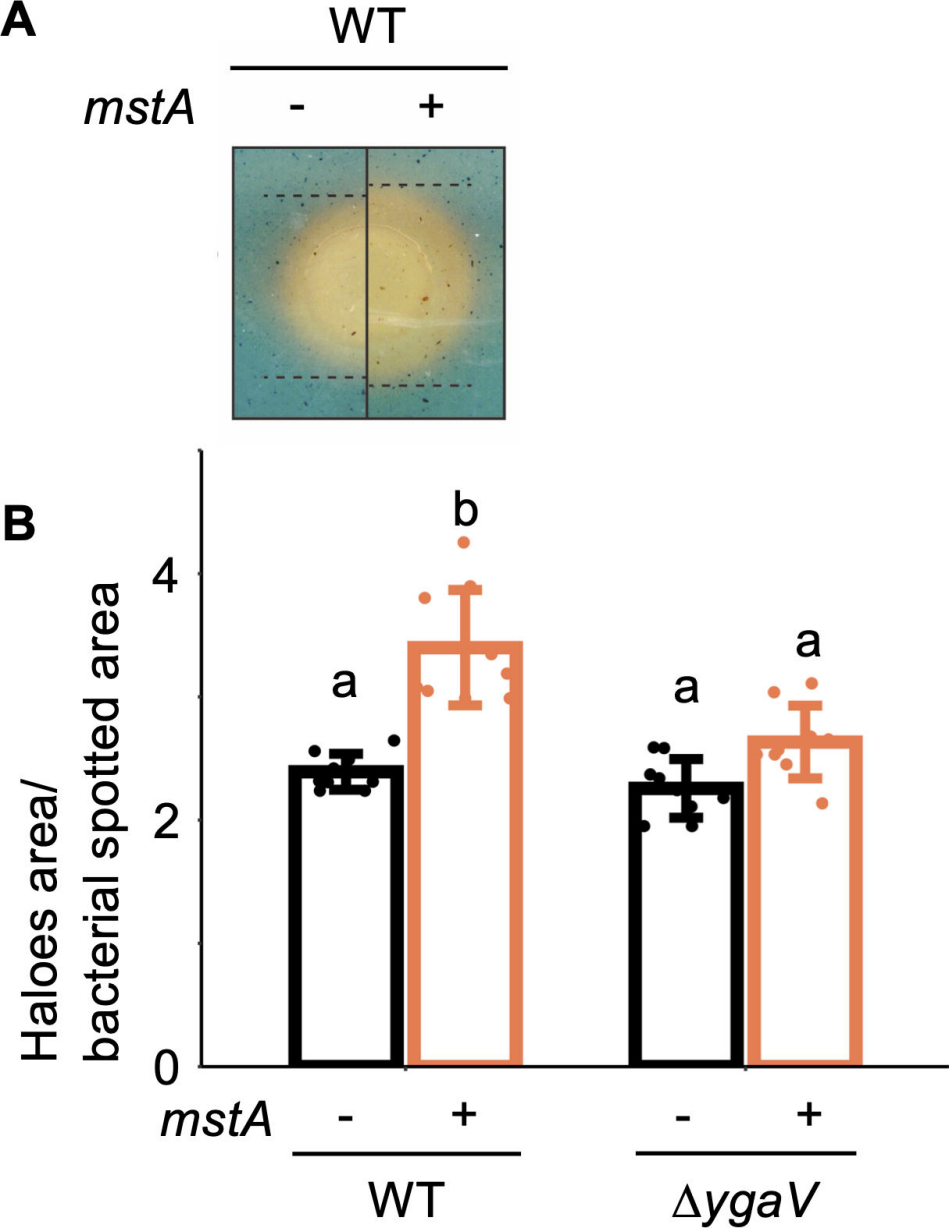
Iron import activity of the *mstA-*overexpressing strain. (**A**) Photographs depicting orange circles formed on CAS agar plates around the spotted bacterial cultures of the WT strain harboring an empty vector (−) and the *mstA*-overexpression plasmid pMstA (+). Dotted lines indicate the boundary of different colors. (**B**) Diameters of the orange circles shown in panel **A**, reflecting the iron transport activity of each strain. The values are means ± SDs of nine biological replicates. Different letters (a and b) indicate a significant difference (*P* < 0.05, Tukey’s test).

Finally, we assessed iron uptake activity of both the WT and Δ*ygaV* strains on chrome azurol S (CAS) agar plate to examine the significance of H_2_S-dependent gene expression control. Free iron manifests as a blue-color pigment on CAS agar plates, allowing estimation of iron uptake activity by measuring the areas of orange zones formed around bacterial culture spots (37). Clearly, WT harboring pMstA exhibited a larger orange hole size compared with the WT carrying pUC18 (Fig. 6A), indicating that the overproduction of intracellular H_2_S induces iron uptake. We also conducted the CAS plate assay with the Δ*ygaV*. Although there was a slight increase in the size of orange holes around the Δ*ygaV* spot upon *mstA* overexpression (Fig. 6B), the difference was not significant. This suggests that the effects of *mstA* overexpression on iron uptake were smaller in the Δ*ygaV* than in the WT, emphasizing the importance of H_2_S-dependent transcriptional control by YgaV for precise regulation of iron uptake activity.

## DISCUSSION

In this study, we explored the impact of hyperaccumulation of intracellular H_2_S on *E. coli* physiology. RNA-seq analysis unveiled significant changes in the gene expression of key genes related to antibiotic resistance, including drug efflux pumps and iron uptake genes, upon overexpression of the *mstA* gene (Fig. 1C; Tables S2 and 3). The H_2_S-responsive transcription factor YgaV is responsible for the upregulation of the iron-uptake gene expression in response to intracellular H_2_S (Fig. 4). Furthermore, iron uptake activity of the Δ*ygaV* strain harboring pUC18 and/or pMstA is the same as that observed in the WT harboring pUC18 (Fig. 6), confirming the importance of YgaV for H_2_S-dependent antioxidative stress responses and antibiotic resistance (11). In *V. cholerae*, intracellular H_2_S level has a positive correlation with iron uptake, which enhances bioavailable iron storage and activates enzymes detoxifying reactive oxygen species such as catalases and a DNA protective protein (5). Increased iron storage was observed in the *mstA*-overexpressing strain (Fig. 3C), supporting the hypothesis that YgaV-dependent transcriptional control is important for H_2_S-dependent enhancement of antioxidative response and antibiotic resistance (11). This further suggests that even if iron limitation occurs in the *mstA*-overexpressing strain due to Fe^2+^ sequestration by FeS formation (4), it is only transient.

Genes involved in heme transport were downregulated in the *mstA*-overexpressing strain (Data Set S1 ). This suggests that intracellular H_2_S synthesis affects heme homeostasis. In several bacteria, heme uptake is modulated in response to iron availability (38). For example, in *Yersinia pseudotuberculosis*, the transcription of the *hmu* operon, encoding a heme acquisition system, is derepressed by Fur under iron-limiting conditions (39). We found that H_2_S overaccumulation, caused by the *mstA* overexpression, increases intracellular iron content (Fig. 3C), suggesting that H_2_S indirectly controls heme homeostasis through iron availability in *E. coli*.

Our previous study showed that YgaV undergoes polysulfidation by sulfide and/or supersulfides to derepress target genes by altering its DNA binding affinity (11), suggesting that polysulfidated YgaV activates the transcription of iron uptake genes (Fig. 5). Interestingly, genes whose expression was altered upon *mstA* overexpression (Data Set S1) did not significantly overlap with those altered by the *ygaV* mutation (11). More specifically, although anaerobic respiratory genes, such as fumarate and nitrate reductase genes, were upregulated in the Δ*ygaV* strain (11), most of these genes were not influenced by *mstA* overexpression (Data Set S1). Furthermore, transcription of the *ygaVP* operon, which is repressed by YgaV in the absence of sulfide (11), was not influenced by *mstA* overexpression (Data Set 1). These results suggest that H_2_S, accumulated due to *mstA* overexpression, could polysulfidate only a limited fraction of YgaV, which may upregulate the target genes such as iron uptake genes, and remaining YgaV could still repress target genes such as those involved in anaerobic respiration. Given that YgaV is a global regulator of redox homeostasis (11), it is also possible that YgaV does not function as a transcriptional activator on its own but requires direct and/or indirect interactions with other transcription factors to upregulate iron uptake genes under *mstA*-overexpression conditions. These hypotheses need to be tested in future studies.

**Fig 7:**
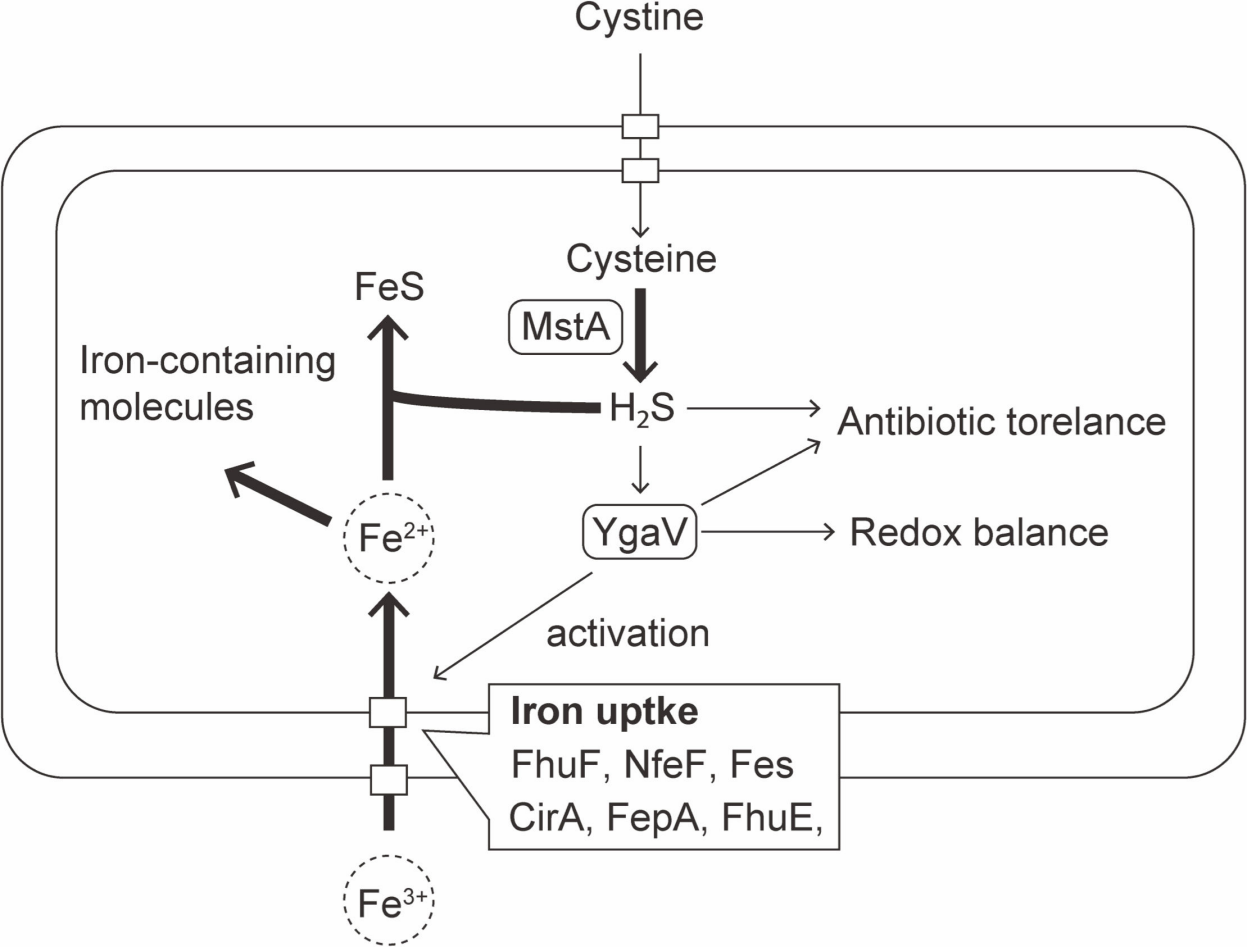
A model illustrating the H_2_S-mediated activation of iron uptake in *E. coli*.

In conclusion, *E. coli* modulates the expression of iron uptake genes via YgaV in response to intracellular H_2_S levels, which is necessary for maintaining iron homeostasis regulated in concert with Fur activity (40, 41). The H_2_S-dependent modulation of gene expression by YgaV contributes to fine-tuning of iron uptake gene expression to increase iron uptake dynamics, crucial for oxidative stress response (42), antibiotic resistance (4, 43), and growth under iron-limiting and sulfide-enriched gut environment (Fig. 7). A more profound understanding of the physiological significance of YgaV-dependent transcriptional control in response to H_2_S holds significant implications for bacterial physiology.

## MATERIALS AND METHODS

### Bacterial strains, growth conditions, and plasmid/bacterial strain construction

All primers used are listed in Table S4. *E. coli* BW25113 served as the WT strain. The Δ*ygaV* mutant was previously constructed from the BW25113 strain (11). The *E. coli* strain expressing His-tagged YgaV, utilized for ChIP-seq analysis, was constructed using the lamda red recombination system (44) with modifications as follows. Initially, a recombinant plasmid containing the tetracycline resistance gene (*tetA*) and the *sacB* gene in tandem was constructed. The *tetA* gene was amplified by PCR using the primer pair tetA-HR_F and tetA-HR_R, with genomic DNA from *E. coli* strain KT1005 containing the Tn10 sequence (45) as a template. The *sacB* gene was amplified using the primer pair sacB-HR_F and sacB-HR_R, with purified DNA of the plasmid pRL250 (46) as a template. The plasmid DNA of pACYC177 (47) was amplified by inverse PCR using the primer pair 177 HR_F and 177 HR_R. Subsequently, the three PCR-amplified DNA fragments were assembled using the Gibson Assembly system (New England Biolabs); the resulting plasmid was named pRIT1. The *tetA-sacB* gene cassette was then amplified by PCR using the primer pair SN1 and SN2, with purified DNA of the plasmid pRIT1 as a template. The amplified fragment contained 50-bp overlap sequences upstream and downstream of the first and second codons of *ygaV*, respectively. The amplified *tetA-sacB* gene cassette was transferred into the *E. coli* WT strain harboring the plasmid pKD46 (44) by electroporation, and tetracycline-resistance colonies were isolated on LB plates as described (44). The double crossover recombination at the target site in the isolated tetracycline-resistant strain was confirmed by colony PCR using the primer pair SN3 and SN4. The His-tag DNA fragment containing 50-bp overlap sequences upstream and downstream of the first and second codons of *ygaV*, respectively, was synthesized by annealing two primers, SN5 and SN6, followed by blunt-ending with KOD DNA polymerase (TOYOBO), and was then transferred into the isolated tetracycline-resistant strain by electroporation. Colonies grown on LB plates containing 15% sucrose were selected as double crossover candidates, and successful integration of the His-tag sequence at the 5′ end of *ygaV* was confirmed by PCR using the primer pair SN3 and SN4, followed by sequencing. Finally, the resulting strain was incubated at 42°C to remove pKD46, and the absence of pKD46 in the strain was confirmed by ampicillin sensitivity testing.

The plasmid for *mstA* overexpression, pMstA, was constructed as follows: initially, the *mstA* gene from BW25113, including Shine–Dalgarno sequence, was amplified by PCR using the primer set SN9 and SN10 (Table S4). The amplified fragment was then cloned into the *Eco*RI-*Hin*dIII cut pUC18 (TaKaRa, Kyoto, Japan), and the resulting plasmid was named pMstA. Introduction of pMstA or pUC18 into *E. coli* strains was achieved by electroporation. *E. coli* strains were cultured in LB medium at 37°C. *E. coli* carrying derivatives of pUC18 were cultivated in LB supplemented with 100 mg/L of ampicillin.

### Quantitative hydrogen sulfide analysis

*E. coli* strains were precultured overnight in LB medium containing ampicillin. These cultures were then diluted to an OD_600_ value of 1.0. The diluted cultures were inoculated into fresh LB medium containing ampicillin at a volume equivalent to 1% of the fresh LB media. These media ware placed on filter paper soaked in lead acetate, positioned 5 mm from the surface of the culture medium in a test tube. The cultures were incubated at 37°C for 24 h with shaking. The brown stain on the lead acetate-soaked paper was quantified using ImageJ software.

### Antibiotic resistance test

A 100-µL aliquot of culture medium with an OD_600_ of 1 was spread on a drug-free LB agar plate. A paper disk containing ampicillin (50 mg/L), gentamicin (50 mg/L), chloramphenicol (50 mg/L), rifampicin (100 mg/L), kanamycin (50 mg/L), streptomycin (50 mg/L), and/or tetracycline (30 mg/L) was placed on the agar plate, followed by incubation at 37°C for 24 h. Subsequently, the diameter of the growth-inhibited zone was measured.

### CAS medium assay

Solid media were prepared with the addition of 1.5% agar. CAS agar plates (37) were employed to assess the secretion of siderophores.

### Quantitation of intracellular iron

Sample preparation for quantitation of intracellular iron followed a previously reported protocol (36). *E. coli* strains were precultured overnight in LB medium containing ampicillin. These cultures were then diluted to an OD_600_ value of 1.0. The diluted cultures were inoculated into fresh LB medium containing ampicillin at a volume equivalent to 1% of the fresh LB media. Cells were cultivated until reaching an optical density (OD) of 0.6. The harvested cells were washed twice with Tris-buffered saline (20 mM Tris–HCl, 150 mM NaCl, pH 8.0) and were braked by sonication for 30 s (sonication every second for 0.5 s, 30 times, amplitude 30%) with a UD-201 ultrasonic homogenizer (TOMY), followed by centrifugation at 17,700 × *g* for 30 min at 4°C. One hundred microliters of supernatant was mixed with 100 µL of 0.1 N HCl and 100 µL of the iron-releasing reagent, a freshly mixed solution of equal volumes of 1.4 N HCl and 4.5% (wt/vol) KMnO_4_ in H_2_O. This mixture was incubated for 2 h at 60°C. After this mixture was cooled at room temperature, 30 µL of iron-detection reagent (6.5 mM ferrozine, 6.5 mM neocuproine, 2.5 M CH_3_COONH_4_, and 1 M ascorbic acid) was added. After 30 min, the absorbance was measured at 550 nm. The iron content of the sample was calculated based on its absorbance, compared to a range of standard concentrations of equal volume that had been prepared in a similar manner to the sample (a mixture of FeCl_3_ standards ranging between 0 and 300 µM was used). The intracellular iron concentration was normalized against the protein concentration of the supernatant.

### RNA-seq analysis

Cells were cultivated in LB medium until reaching an OD of 0.6, and total RNA was extracted from the harvested cells using the RNAprotect Bacteria Reagent (Qiagen), followed by the RNeasy Mini Kit (Qiagen). The total RNA (1 µg) was depleted of ribosomal RNA (rRNA) using the QIAseq-FastSelect-5S/16S/23S Kit (Qiagen). Indexed RNA-Seq libraries were prepared from the rRNA-depleted RNA samples using the NEBNext Ultra II Directional RNA Library Prep Kit for Illumina (New England Biolabs) according to the manufacturer’s instructions. Libraries were sequenced using the Illumina NovaSeq 6000 platform to obtain 101-bp single-end reads (13.3 ± 0.6 million reads/sample). Sequence reads were analyzed using the ProkSeq v.2.0 pipeline (21), which employs FastQC and AfterQC for trimming and quality control, Bowtie2 for sequence alignment, FeatureCounts for the quantification of RNA expression levels, and DEseq2 for differential expression analysis. Genes were considered as being differentially expressed when their adjusted *P* values and absolute log2 fold changes were <0.05 and >1.0, respectively. The RNA-seq experiment was repeated using three independent biological replicates.

### Quantitative real-time PCR

Cells were cultivated in LB medium until reaching an OD of 0.6, and total RNA was extracted from the harvested cells using the RNAprotect Bacteria Reagent (Qiagen), followed by the RNeasy Mini Kit (Qiagen). qRT-PCR was performed using the total RNA as templates with PrimeScript RT regent kit (TaKaRa) on a Thermal Cycler Dice Real-Time System III (TaKaRa). Primers used are described in Table S4. The qRT-PCR was repeated using three independent biological replicates.

### ChIP-qPCR analysis

Sample preparation for ChIP-qPCR analysis followed a previously reported protocol (48). Briefly, His-tagged YgaV-expressing *E. coli* strains, harboring pUC18 or pMstA, were precultured overnight in LB medium containing ampicillin. These cultures were then diluted to an OD_600_ value of 1 and inoculated into fresh LB medium containing ampicillin at a volume equivalent to 1% of the fresh LB media. Cells were cultivated until reaching an OD of 0.6 and then fixed with 1.2% formaldehyde. Cells were treated with 20-mg/mL lysozyme in 2 mL PeriPrep buffer containing 200 mM Tris–HCl (pH 8.0), 50% (vol/vol) sucrose, 1 mM phenylmethylsulfonyl fluoride (PMSF), and 1% (vol/vol) proteinase inhibitor cocktail (Nacalai Tesuque). After incubation for 20 min at 37°C with shaking, cells were centrifuged at 7,000 × *g* for 5 min at 4°C, and then resuspended in 550 µL King2 buffer containing 100 mM Tris–HCl (pH 7.5), 200 mM NaCl, 1% (vol/vol) Triton X-100, 0.1% (wt/vol) Na-deoxycholate, 0.2% (wt/vol) Brij 58, and 20% (vol/vol) glycerol. DNase I treatment was performed with 50 µL of MgCa buffer containing 100 mM MgCl_2_ and 50 mM CaCl_2_, 200 µg RNase A, and 20 units of DNase I at 37°C for 15 min. Reactions were stopped by adding 3 mL of UT buffer containing 50 mM HEPES (pH 7.6), 250 mM NaCl, 0.5% (vol/vol) Triton X-100, 40 mM imidazole, 5 mM β-mercaptoethanol, 9 M urea, 1 mM PMSF, and 1% (vol/vol) proteinase inhibitor cocktail. The resultant suspensions were sonicated for 30 sec (0.6 s “on”/0.4 s “off,” 30 times, amplitude 30%) with a UD-201 ultrasonic homogenizer (TOMY), followed by centrifugation at 12,000 × *g* for 5 min at 4°C. Half of the supernatant was mixed with Ni-charged His-binding resin (Millipore), and the remaining half was mixed with Ni-uncharged His-bind resin as a negative control. The mixtures were rotated overnight at room temperature, and the resins were washed seven times with UT Buffer, and resuspended in 250 µL of elution buffer containing 50 mM HEPES (pH 7.6), 250 mM NaCl, 0.5% (vol/vol) Triton X-100, 500 mM imidazole, 5 mM β-mercaptoethanol, and 9 M urea. One hundred forty microliters of the elution samples was mixed with 60 µL of M-Wash Buffer containing 0.1 M Tris–HCl, pH 7.5, 1% (wt/vol) SDS, and 10 mM dithiothreitol, followed by incubation at 65°C for 6 h for decrosslinking. Proteins were removed by phenol–chloroform–isoamyl alcohol extraction, and DNA was purified by ethanol precipitation. Finally, ChIP DNA was analyzed by qPCR using specific primer sets (Table S4).

### Statistical analysis

Statistical analysis was performed using Microsoft Excel for Welch’s *t*-test and a Python-based homemade program for Tukey’s test. The sample size (*n*) and the nature of replicates have been provided wherever relevant.

## Data Availability

The results of the RNA-seq experiments are available in Data Set S1, and their raw data have been deposited in the DDBJ database under the accession number DRA016725.

## AFTER EPUB

*[This article was published on 26 September 2024 with an error in Importance. The error was corrected in the current version, posted on 1 October 2024.]*

## SUPPLEMENTAL MATERIAL

Data Set S1 mbio.01991-24-s0001.xlsx Raw data of the RNA seq.

Fig. S1 mbio.01991-24-s0002.pdf Phenotype of the *mstA*-overexpressing strain in the absence of antibiotics.

Table S1 mbio.01991-24-s0003.xlsx tRNA synthase genes whose expression are upregulated in the *mstA*-overexpressing strain.

Table S2 mbio.01991-24-s0004.xlsx Antibiotic efflux transporter genes whose expression are upregulated in the *mstA*-overexpressing strain.

Table S3 mbio.01991-24-s0005.xlsx Iron uptake genes whose expression are upregulated in the *mstA*-overexpressing strain.

Table S4 mbio.01991-24-s0006.xlsx Primers used in this study.

ASM does not own the copyrights to Supplemental Material that may be linked to, or accessed through, an article. The authors have granted ASM a non-exclusive, world-wide license to publish the Supplemental Material files. Please contact the corresponding author directly for reuse.
