# Supplementary material for: Increased intracellular H2S levels enhance iron uptake in Escherichia coli
Source: mBio. 2024 Sep 26;15(10):e01991-24. doi: 10.1128/mbio.01991-24 (PMC11481527; doi:10.1128/mbio.01991-24)
Supplement: Fig. S1 — Phenotype of the mstA-overexpressing strain in the absence of antibiotics. [file mbio.01991-24-s0002.pdf]

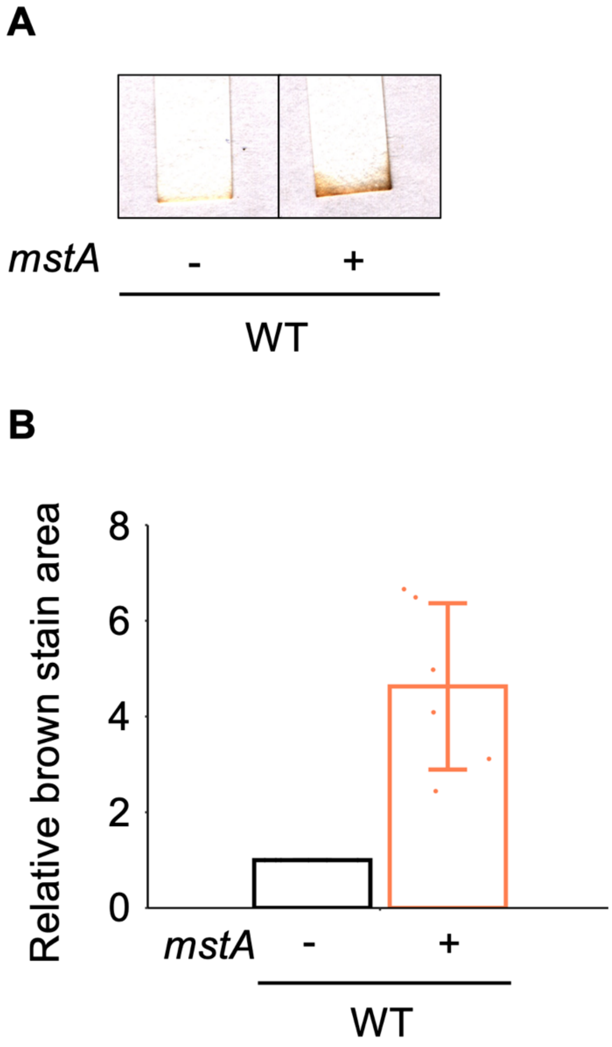

Fig. S1 Phenotype of the *mstA*-overexpressing strain in the absence of antibiotics. (A) Brown staining on $Pb(Ac)_2$-soaked papers reflecting H_2S synthesis in the WT strain harboring the empty vector (−) or the *mstA*-overexpression plasmid pMstA (+) in the absence of antibiotics. (B) Quantification of brown-stained areas on $Pb(Ac)_2$-soaked papers shown in (A). The values are means ± SD of six biological replicates. $^{**}p < 0.05$, Welch's *t*-test.
